# Supplementary figures and images for: Bacterial surface interactions with organic colloidal particles: Nanoscale hotspots of organic matter in the ocean
Source: PLoS One. 2022 Aug 25;17(8):e0272329. doi: 10.1371/journal.pone.0272329 (PMC9409529; doi:10.1371/journal.pone.0272329)

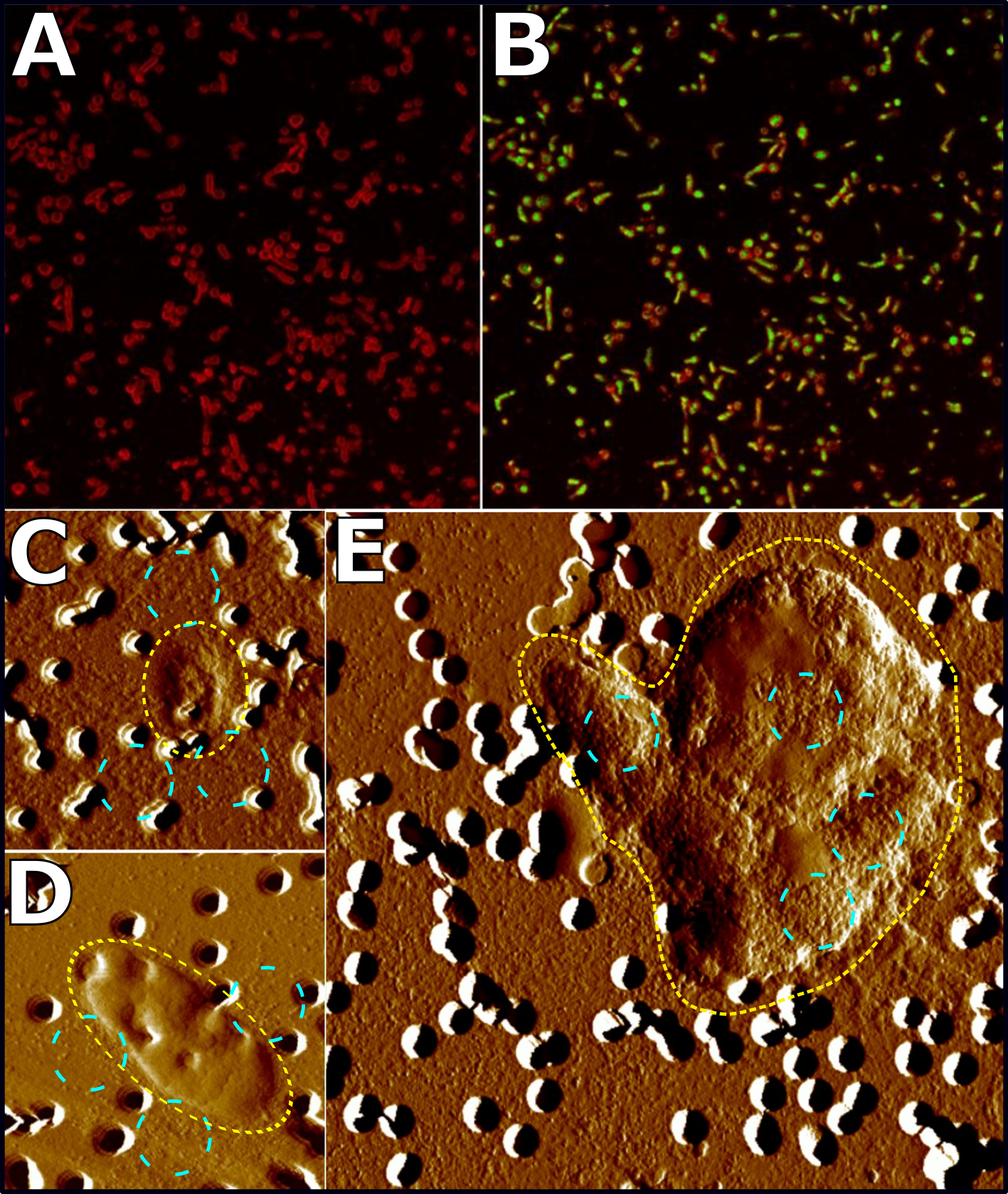

Supplement: S1 Fig — Fluorescence images show bacterial cells (red: FM 4-64fx) variably covered with ribosome (green: SYBR Green II), when amended to a ribosome concentration of 5 × 10^9^ particles mL-1 (before (A) and after (B) 60s ribosome amendment). AFM peak force error images of cells from 0.6-μm-filtrate (C) and a marine isolate Alteromonas sp. ALTSIO cell (D) are associated with small films or clusters of ribosome particles (dashed regions). Cells were observed after ribosome amendment 3.3 μg mL-1 (= 8 × 10^11^ particles mL-1). (E) AFM peak force error image showing a cell from natural assemblage with the surface covered by large patches of particles (dashed regions) after amendment and exposure to ribosomes. (TIF) [file pone.0272329.s001.tif]

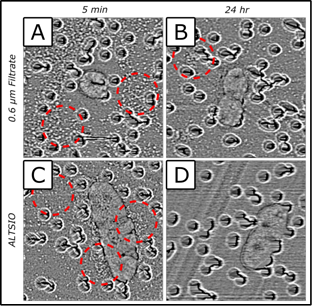

Supplement: S2 Fig — AFM imaging study of bacterial depletion of added ribosomes. Ribosomes were amended to bacterial isolate ALTSIO and 0.6-μm-filtrate natural assemblage cells and incubated for 24 h. Results show the depletion of ribosomal particles (red dashed regions) for 0.6-μm seawater filtrate cells (A, B) and Alteromonas sp. ALTSIO cells (C, D). Mean curvature images processed from topographic data show fewer ribosome particles (white particle features) on the background 0.22-μm polycarbonate filter at 0 h, minutes after amendment, (A, C) and after 24 h incubation (B, D). (TIF) [file pone.0272329.s002.tif]

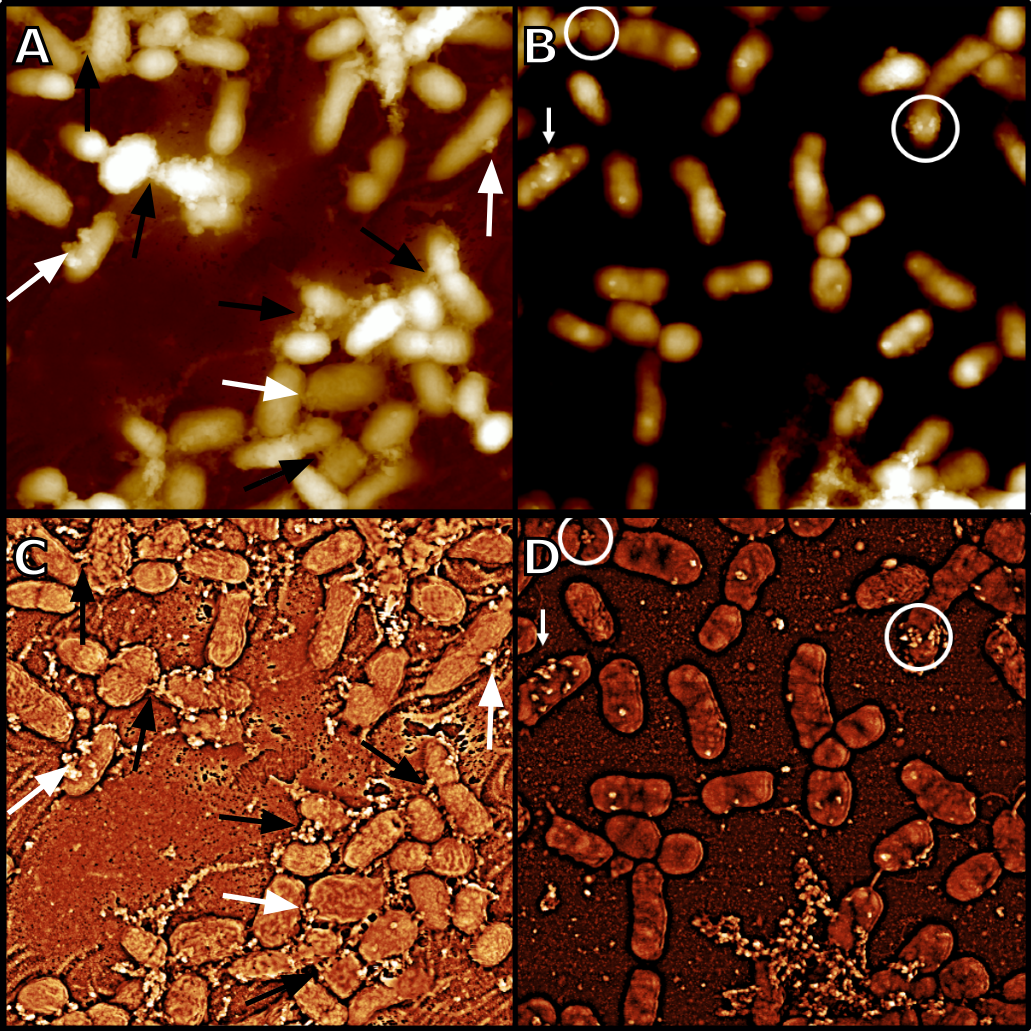

Supplement: S3 Fig — AFM (A, B) and respective SEM presentations (C, D) of Alteromonas sp. ALTSIO cells. Many cells have substantial surface patches of ribosomes after extended amendment times (4.5 h) to high concentrations. Example surface patches are indicated by white arrows and white circles. In certain regions, surface patches appear to coalesce into larger patches, towards forming a contiguous film of particles that covers a group of cells. Panel image scan sizes are 10 μm × 10 μm. (TIF) [file pone.0272329.s003.tif]

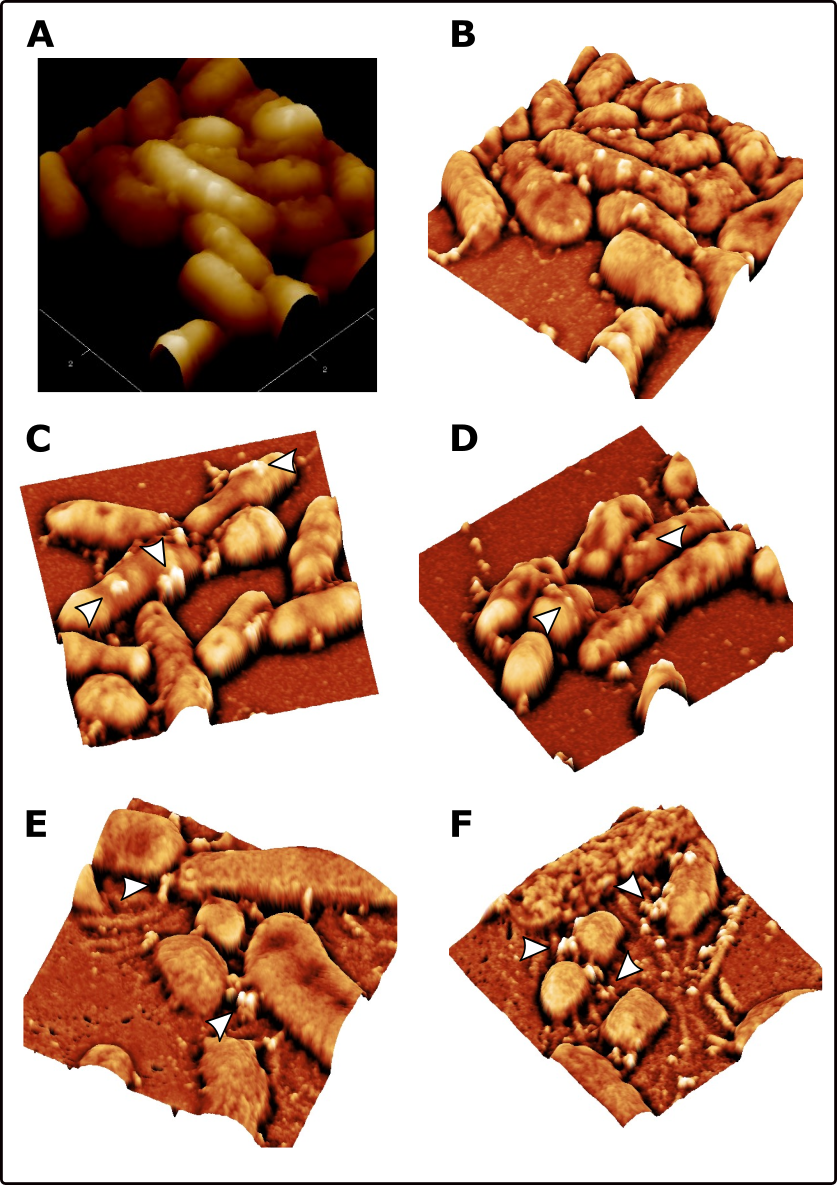

Supplement: S4 Fig — 3D surface representations of AFM topographic data (A) and respective SEM presentations (B) of an Alteromonas sp. ALTSIO cell with multiple surface particle clusters (white protruding features). (C-F) SEM presentation images showing different regions and features of ribosome attachment on bacterial surfaces, where small groups of particles attach directly onto bacterial surfaces (C, D) or indirectly to cells (E, F). (TIF) [file pone.0272329.s004.tif]

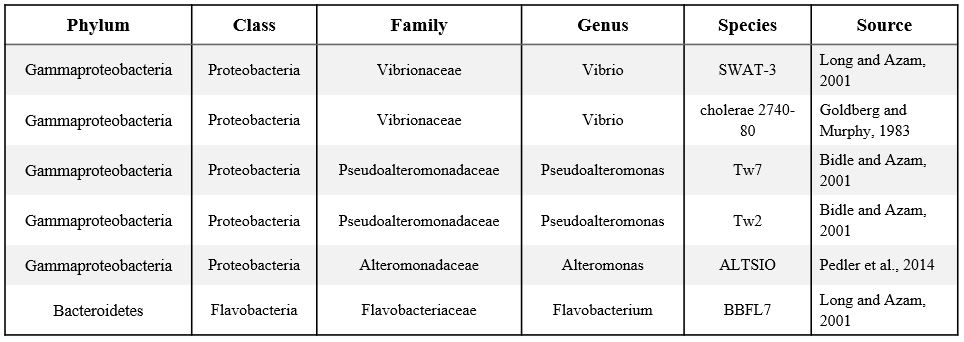

Supplement: S1 Table — (TIF) [file pone.0272329.s005.tif]
