# Supplementary material for: Bacterial surface interactions with organic colloidal particles: Nanoscale hotspots of organic matter in the ocean
Source: PLoS One. 2022 Aug 25;17(8):e0272329. doi: 10.1371/journal.pone.0272329 (PMC9409529; doi:10.1371/journal.pone.0272329)
Supplement: Attachment — Submitted filename: Review of PONE-D-22-10338 .pdf [file pone.0272329.s006.pdf]

Review of PONE-D-22-10338

This paper on 'Bacterial surface interactions with organic colloidal particles: nanoscale hotspots of organic matter in the ocean' is well written and articulated, and I recommend publication with minor changes.

General comments

For greater relevance it might be good to have a statement which compares conditions and concentrations in the experiments to those in natural seawater, e.g., the relative concentrations and ratio of ribosome colloids to bacteria.

Detailed comments

Line 522: 'Isolate cultures' should be 'Isolated cultures'

Line

Line 676 and 679: What is 'AFM error mode'?. Please explain

Line 683: 'Ribosomes were amendment' should be 'Ribosomes were amended'

Line 689: What is 'SEM-like presentations '? Please explain

Line 697: What is 'SEM simulated image'? Please explain
