# Supplementary material for: Bacterial surface interactions with organic colloidal particles: Nanoscale hotspots of organic matter in the ocean
Source: PLoS One. 2022 Aug 25;17(8):e0272329. doi: 10.1371/journal.pone.0272329 (PMC9409529; doi:10.1371/journal.pone.0272329)
Supplement: Attachment — Submitted filename: Response to Reviewers.docx [file pone.0272329.s007.docx]

Dear Editor,

We appreciate you and the reviewers for your time in reviewing our paper and providing valuable comments. The insightful comments and helpful feedback have led to improvements in the current version. We have uploaded the missing Supplementary Table 1 as requested. The minimal dataset is available at figshare (<https://doi.org/10.6084/m9.figshare.20304786.v1>). The authors have carefully considered the comments and have tried our best to address every one of them. The authors welcome further constructive comments if any. Below we provide the point-by-point responses. All modifications in the revised manuscript have been highlighted.

Sincerely,
Nirav Patel

**Review Comments to the Author:**

Reviewer #1: This paper on ‘Bacterial surface interactions with organic colloidal particles: nanoscale hotspots of organic matter in the ocean’ is well written and articulated, and I recommend publication with minor changes.

General comments
For greater relevance it might be good to have a statement which compares conditions and concentrations in the experiments to those in natural seawater, e.g., the relative concentrations and ratio of ribosome colloids to bacteria.
A statement has been included to compare the concentrations of ribosomes in seawater to the concentrations used in experiments as follows: “Extracellular ribosomes in ambient seawater, from 16S rRNA proxy measurements, are present at approximately 10^7^ ribosomes mL^-1^ , and can reach elevated concentrations up to 10^9^ ribosomes mL^-1^ in regions enriched after cell lysis” (line 114)

(**NOTE**: The line numbers referred to correspond to the “Revised Manuscript with Track Changes” file, not the unmarked-up file “Manuscript”.

Detailed comments
Line 522: ‘Isolate cultures’ should be ‘Isolated cultures’
Corrected (line 548)

Line 676 and 679: What is ‘AFM error mode’?. Please explain
The term “AFM error mode” was updated in the manuscript to “peak force error” for accuracy. A brief explanation of the “peak force error” imaging has been included (line 573). AFM error mode images are a fundamental part of atomic force microscopy where images are generated from the tracking error of tip movement. The text was updated to state that peak force error images are generated from setpoint error of applied peak forces during AFM images.

Line 683: ‘Ribosomes were amendment’ should be ‘Ribosomes were amended’
Corrected (line 752)

Line 689: What is ‘SEM-like presentations ’? Please explain
Line 697: What is ‘SEM simulated image’? Please explain
We corrected the inconsistent terms of “SEM-like presentations” and “SEM simulated image” to “SEM presentations”. A brief statement was included to explain the processing of AFM height images into SEM image presentations of the topographic data for visualization purposes (line 575).

Reviewer #2: The manuscript demonstrated bacterial surface interactions with nano-sized colloidal particles including colloid-sized surface corrugations and protrusions. The data showed that ribosomes can attach onto bacterial surfaces and bacteria can readily utilize this organic matter as substrates to growth. This study provided useful information about the adaptive strategies of pelagic marine bacteria for colloid capture and utilization as nutrients. Overall, this study is interesting and easy to follow. Comments are listed in the bellow:
1. Line 33: the unit of organic colloid abundance should be “particles mL-1”
Corrected (line 36)

2. The manuscript lacks statistic data, only providing AFM image looks weak. Please added some statistic data or figure form height, thickness and size at different experiments and further compared them.
Our study resulted in qualitative data for describing the outcomes of bacterial surfaces exposed to colloidal particles. The collected data was primarily qualitative in denoting the presence/absence of surface features from attached particles. Quantitative description of these features was difficult in many cases, including cells with excessive particle attachment and in regions with touching or overlapping bacteria. In such cases, particles could not be adequately characterized compared to more prominent counterparts of one or two particles or clusters on an individual cell. This contributed to difficulties in characterizing particle attachment in an unbiased manner for statistical comparisons. Future studies could further explore statistical comparisons of features and observations based on the limited qualitative descriptions.

3. Did you consider measuring attached forces with AFM, the force measurements will help explain mechanisms of surface corrugations, attachment, and protrusions.
Our current study was primarily focused on nanoscale changes of bacterial surfaces after exposure to organic colloids. We did consider investigating the attachment forces of individual particles to collect data that may help explain mechanisms of particle attachment. Such studies will help explain mechanisms of initial attachment events of individual particles on bare bacteria and may be limited for surface clusters and aggregates as discussed in the manuscript. Such studies would involve applying AFM force spectroscopy to probe interaction forces of individual particles in constrained configurations and conditions and may not reflect the range of microenvironmental conditions experienced by individual bacteria interacting with colloidal particles.

4. Many references are missing in the manuscript, need citing references to support the discussion.
References have been added to support various discussion statements (line 367-375, 431-438, 448-452)

5. Line 283 which physical properties? Should further discuss
We have included a brief statement regarding the physical properties as follows: “Physical properties, such as charge distribution, hydrophobicity and surface roughness can influence the interaction forces of individual particles with bacterial surfaces [45-48]. The spatial distribution of charged residues and hydrophobic structures on bacterial surfaces may contribute to the attractive forces promoting the retention and localized clustering of surface particles in specific regions.” (line 289)

6. Line 443-450, need reference to support.
References have been added to support discussion statements (line 462-476)
